# Review of Research on Road Traffic Operation Risk Prevention and Control

**DOI:** 10.3390/ijerph191912115

**Published:** 2022-09-25

**Authors:** Yongji Ma, Jinliang Xu, Chao Gao, Minghao Mu, Guangxun E, Chenwei Gu

**Affiliations:** 1 School of Highway, Chang’an University, Xi’an 710064, China; 2 Shandong Hi-Speed Group Co., Ltd., Jinan 250098, China

**Keywords:** traffic engineering, road traffic risk prevention and control, review, human factor risk, traffic risk

## Abstract

Road traffic safety can be ensured by preventing and controlling the potential risks in road traffic operations. The relevant literature was systematically reviewed to identify the research context and status quo in the road traffic operation risk prevention and control field and identify the key study contents needing further research. As research material, the related English and Chinese literature published between 1996 and 2021 (as of 31st December 2021) was obtained through the Web of Science Core Collection and Chinese Science Citation Database. These research materials include 22,403 English and 7876 Chinese papers. Based on the bibliometrics, this study used CiteSpace software to conduct keyword co-occurrence analysis in the field. The results show that the relevant research topics mainly covered the risks of drivers, vehicles, roads, and the traffic environment. In the aspect of driver risks, the studies focused on driving behavior characteristics. In terms of vehicle risks, the related studies were mainly about the vehicle control system, driving assistance system, hazardous material transportation, automated driving technology, safe driving speed, and vehicle collision prediction. For the road risks, the safe driving guarantee of high-risk road sections, driving risks at intersections, and safe road alignment design were the three study hotspots. In terms of traffic environment risks, identifying traffic risk locations and driving safety guarantees under adverse weather conditions were the two main research highlights. Moreover, mathematical modeling was the main method for studying road traffic operation risk. Furthermore, the impact of environmental factors on drivers, the emergency rescue system for road traffic accidents, the connection between automated driving technology and safe driving theory, and the man–machine hybrid traffic flow characteristics are the subjects needing further research.

## 1. Introduction

Road traffic accidents often cause property losses; these accidents even endanger the lives of road users. Effective prevention and control of the road traffic operation risks and ensuring road traffic safety have always been complex problems for many countries. World Health Organization and the United Nations (UN) regional commissions jointly issued the Global Plan for the Decade of Action for Road Safety 2021–2030 [1] in cooperation with other partners in the UN Road Safety Collaboration. According to this global plan, road traffic accidents kill nearly 1.3 million people and injure nearly 50 million people every year worldwide. Moreover, the global road traffic accidents in the next decade may cause approximately 13 million deaths and 500 million injuries if the existing road traffic operation risk prevention and control system cannot be optimized and upgraded. In China, more than 60,000 and 200,000 people are killed and injured in traffic accidents every year, respectively, from 2014 to 2021 [2]. Thus, road traffic safety has become a key issue affecting sustainable development.

Road traffic safety is a system engineering problem comprising drivers, vehicles, roads, road ancillary facilities, driving environment, and traffic management [3,4,5]. It is affected by driving behaviors, vehicle and road characteristics, and driving conditions. On the one hand, road traffic safety can be viewed as a design problem for vehicles and roads. On the other hand, it can be seen as a matter of road operations, driving habits, traffic rules, enforcement, and risk management. Road traffic safety is closely related to the whole field of transportation. Thus, ensuring road traffic safety only through the strength of several aspects is insufficient.

We can regard the road traffic operation risk as the occurrence probability of traffic accidents to a certain extent. Traffic risks constantly exist regardless of the prevention and control measures taken; that is, the occurrence probability of accidents would never be zero because of the complexity of the road traffic system. The key to solving the road traffic safety problem lies in the in-depth study of the hidden safety risks in each link of the road traffic system. Moreover, this problem can be solved by exploring the macrolevel and microlevel of driving risks and taking the corresponding prevention and control measures to reduce the occurrence probability of all kinds of traffic accidents. Therefore, the causes and transmission characteristics of risks must be studied. Furthermore, rapid response and emergency rescue should be performed when traffic accidents occur to minimize the loss of lives and property of relevant personnel.

Considerable research worldwide roughly followed the ideas mentioned above in terms of contents and technical routes. 

For example, studies on drunk driving [6,7], fatigue driving [8,9], distracted driving [10,11], and the characteristics of elderly drivers and young drivers [12,13] are subject to the research area of human factor risks. Related results showed that alcohol would cause the lag of driver’s response and the reduction of driver’s action ability; fatigue would lead to the driver’s inattention, miscalculation, and neglect of driving conditions; degradation of physiological indexes of elderly drivers would also reduce their driving ability. These factors are the main threats to road traffic safety. 

Studies themed on driver assistance system [14], vehicle collision risk prediction and assessment [15,16], vehicle-lane-changing safety [17,18], and risk prevention and control of hazardous material transportation vehicles [19,20] belong to the research area of vehicle factor risks. Representative achievements of relevant research mainly include the design theory of driving assistance system, collision avoidance algorithm and model of autonomous vehicles, vehicle-lane-changing risk assessment, and decision-making technology. All of which have laid the foundation for improving the safety and intelligence of vehicles. They also promote the construction of intelligent transportation system (ITS). 

Studies on road alignment safety [21], climbing lane safety [22], and other topics belong to the research area of road factor risks. The relevant research obtained the results of road safety design indicators under different working conditions. These results have laid a theoretical foundation for improving road design standards. 

Studies on driving safety in rainy, snowy, and foggy weather conditions [23,24] belong to the research area of traffic environmental factor risks. Relevant results have further deepened people’s understanding of vehicle driving and traffic flow characteristics under adverse weather conditions. These results have also laid a foundation for improving the theory of road traffic flow. Some researchers conducted research from the perspective of the entire process of road traffic risk management, which involves many aspects, such as perception [25,26], assessment [27,28], prediction [29], evolution [30], and avoidance [31] of driving risk.

In summary, the research on road traffic operation risk prevention and control has covered extensive areas and yielded successful results. The present study analyzed the literature research materials, combined the development context and research results, compared the relevant technical characteristics, and summarized the research priorities and complex problems in the field by using the bibliometrics method through CiteSpace software. On this basis, the future research direction and focus of road traffic operation risk prevention and control were clarified.

## 2. Materials and Methods

This section mainly introduces the materials and methods used in this study, including the review method, literature retrieval databases and keywords, literature screening method, and literature retrieval and screening results.

### 2.1. Review Method

The review of road traffic safety involves representative research methods, mainly including narrative review and meta-analysis methods.

In general, most researchers mainly adopt the narrative review method when conducting a review in a certain field. The narrative review method is a traditional and representative review method. It is also the main method in the road traffic field [32,33,34]. This method is usually used to collect relevant literature around a certain topic according to specific purposes, needs, and interests. It is also used to analyze and evaluate the research content of a paper by using qualitative analysis methods. Moreover, it is used to elaborate and comment on the paper according to personal views and experience. Next, the research content is summarized in writing. Narrative review can provide a large amount of new knowledge and information for a certain field or specialty, facilitating readers to understand the general situation and development direction of particular research in a relatively short time. Next, this method solves the problems encountered in practice. It is applicable to the review of almost all traffic safety problems. The collection and sorting of review materials are mainly performed by manual retrieval and reading; thus, the workload is large. The narrative review method particularly applies to detailed research on issues with a small coverage.

Given the gradual promotion and application of the meta-analysis method, some researchers in the transportation field began to use it to summarize and study a specific problem in the road traffic safety field [35,36,37]. Meta-analysis is a statistical method that combines and analyzes multiple results with the same research purpose. It can answer relevant research questions with high test efficiency under a large sample size. Meta-analysis uses quantitative statistical methods to analyze, synthesize, and summarize the existing research results. It belongs to quantitative systematic evaluation and is recognized as one of the best secondary research methods. It is applicable to the research of specific problems and has high requirements for the chosen references. In general, the chosen references should be empirical research, the data in the paper should be complete, and the sample size should be clear. Moreover, pure theory and review articles should be excluded.

On the one hand, the research theme of road traffic operation risk prevention and control has extensive coverage and involves very complex contents. Thus, using meta-analysis to summarize and study this research theme is difficult, given the macrolevel and microlevel factors. On the other hand, collecting the literature by pure manual reading and screening is difficult because of the complexity and diversity of the research literature involved. Therefore, we finally adopted the narrative review method and assisted the literature screening work with software to improve the efficiency and accuracy of literature screening.

### 2.2. Literature Retrieval Databases and Keywords

The Web of Science Core Collection (WoSCC) and Chinese Science Citation Database (CSCD) were chosen as the literature retrieval databases in the present study. Moreover, the search keywords from the perspectives of risk type, risk source, and risk prevention and control research were determined according to the connotation and extension of road traffic operation risk prevention and control. The chosen keywords at each level should be the most representative terms in the road traffic field, the key objects of road traffic safety research, and the most concerning risk prevention and control field of traffic management departments to obtain comprehensive and accurate review materials. Therefore, on the basis of our understanding of road traffic and the suggestions of relevant experts in the industry, we finally determined the following keywords as the basis for retrieval:

1. Keywords at the risk type level: Driving risk, traffic risk, traffic conflict, collision risk, roller risk, sideslip risk, rear-end collision, and road risk.
2. Keywords at the risk source level: Driving behavior, alcohol driving, drug driving, distracted driving, fatigue driving, aggressive driving, elderly drivers, novice drivers, dangerous vehicle, dangerous-goods truck, hazardous material transportation, dangerous section, tunnel section, bridge section, long downhill, sharp bends section, road design, horizontal curve, vertical curve, poor traffic environment, adverse traffic weather, rain, foggy weather, and snow.
3. Keywords at risk prevention and control research level: Risk evolution, risk identification, risk perception, risk assessment, risk prediction, risk behavior monitoring, risk management, risk correction, risk avoidance, risk response, road emergency, traffic emergency, traffic control, cooperative vehicle infrastructure system, road infrastructure, automatic driving, vehicle guidance, traffic guidance, and speed control.

From the perspective of precision and recall of literature retrieval, the more specific the keyword restrictions are, the higher the precision is and the lower the recall of the search results are. Moreover, the broader the keyword setting is, the lower the precision is and the higher the recall is. Some documents unrelated to the research topic are inevitably mixed into the search results during literature retrieval. Therefore, considering the recall and precision of literature retrieval, we added traffic, road, highway, and vehicle to refine the literature search results for the aforementioned keywords lacking the theme of road traffic. Similarly, risk and safety were supplemented to refine the literature search results for keywords lacking the risk theme. In addition, the disciplines, topics, and other options in each database were used to screen the literature.

### 2.3. Literature Screening Method

This study used the software CiteSpace as a tool for extracting the knowledge information from the literature to mine the knowledge information contained in the massive, collected literature deeply and efficiently. CiteSpace is a literature exploration software developed by Chen CM [38,39]. Thomas Kuhn’s theory of scientific development mode, Pune’s scientific frontier theory and structural holes theory, Klein Berger’s burst detection technology, the best foraging technology for scientific communication, and the theory of knowledge unit dispersion and reorganization are the main ideological foundations of CiteSpace. Based on co-citation analysis theory and the path-finding network algorithm, CiteSpace can realize the in-depth mining of the literature information of a certain discipline; it is also the interdisciplinary application of multiple disciplines, such as bibliometrics, scientometrics, and informetrics [39]. 

The software can sort out and integrate the information, knowledge structure, and logical correlation of massive documents more objectively, scientifically, and efficiently than reading and summarizing scientific literature information manually. Hence, CiteSpace has been applied in the review of many different subjects [40,41]. 

The keywords in the literature can represent the main research directions, and those with high frequency represent the hotspots, key points, and difficult issues in the research field. Using keywords can help us grasp the research context and progress in the professional field and further clarify the future research direction. Hence, the present study mainly used the keyword co-occurrence analysis function of CiteSpace to extract from the extensive literature the important keywords and the key papers to which they belong. Next, the research actuality of road traffic operation risk prevention and control was explored by using them as the main review materials. When determining the key papers according to the keywords, we mainly used the citation number ranking of the papers as the basis. We also focused on the papers with high citation times.

### 2.4. Literature Retrieval and Screening Results

We set the retrieval time spans of the WoSCC and the CSCD as “all years (1985–2021)” and “all years (1989–2021)”, respectively. Next, the literature retrieval was performed according to the determined strategy. We selected the literature types as article, review, and proceedings paper. We also made full use of the discipline and theme refining function of the databases and further combined manual screening to eliminate the literature related to railways, water transportation, aviation, investment, environmental protection, medicine, and other irrelevant topics. Moreover, we used the record deduplication function of CiteSpace to eliminate duplicate documents. Finally, the number of papers in the field of road traffic operation risk prevention and control in each database is as follows:

1. WoSCC: A total of 22,403 papers were retained from 1996 to 2021 after deduplication.
2. CSCD: A total of 7876 papers were retained from 1999 to 2021 after deduplication.

For academic papers, in addition to the keywords specifically given by the authors, other terms related to the topic of the study in the abstract and title sections can usually provide clues for us to obtain the research information of the paper. Thus, this study chose the keywords in the broad sense. In particular, apart from the keywords given in the research papers, important professional terms in the road traffic operation risk prevention and control field were extracted from the title and abstract of the paper as keywords for co-occurrence analysis to avoid omitting important terms that did not appear in the keyword display section of the literature. The papers in each database were segmented at 2-year intervals when CiteSpace was used to extract keyword co-occurrence analysis data. Moreover, 200 papers with the top citations in each period were selected as analysis materials. All of the works from periods with less than 200 papers in 2 years were taken.

## 3. Results

### 3.1. Literature Review of WoSCC

**Figure 1:**
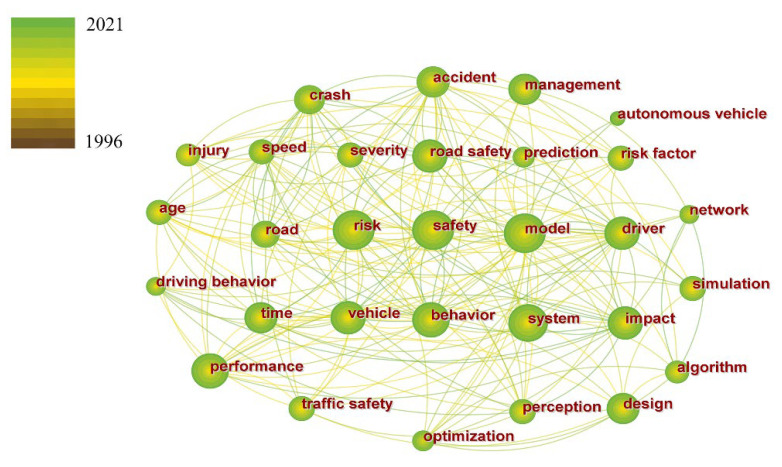
Co-occurrence network of partial high-frequency keywords in WoSCC.

For the 22,403 relevant papers in WoSCC, 1234 keywords that met the threshold conditions were extracted by CiteSpace. Figure 1 shows the co-occurrence network of partial high-frequency keywords in WoSCC.

In Figure 1, the node size indicates the occurrence frequency of the keyword. The larger the node is, the greater the occurrence frequency of the keyword is, and vice versa. The node’s color indicates the published time of the paper to which the keyword belongs. A keyword node contains different colors, indicating that the keyword was used in papers published in different years. The line between nodes indicates that the group of keywords has appeared in the same paper, and the color of the line corresponds to the published year of the first paper where the keywords are located.

According to the frequency ranking, the top 30 keywords in the 26 years from 1996 to 2021 are as follows:

Model, risk, safety, system, behavior, impact, performance, design, driver, vehicle, management, accident, crash, time, road, injury, speed, perception, simulation, prediction, network, algorithm, age, optimization, framework, severity, collision, risk factor, traffic flow, and identification.

#### 3.1.1. Analysis of “Model”

**Figure 2:**
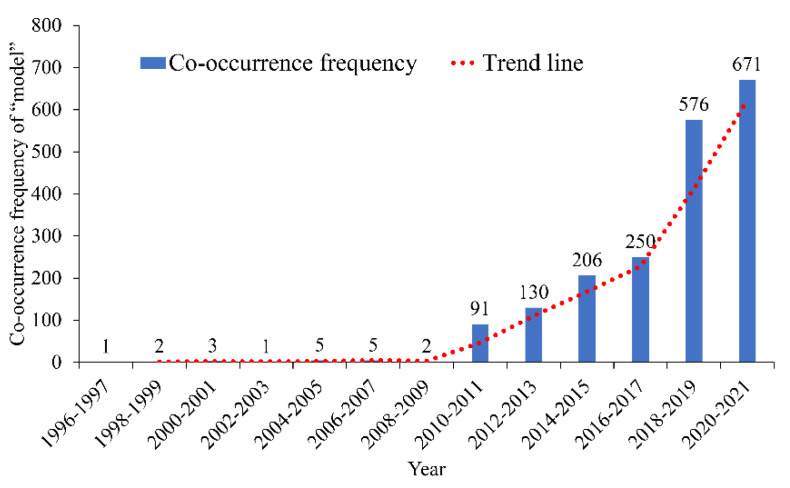
Co-occurrence frequency and time distribution of “model”.

Among all keywords, “model” has the highest co-occurrence frequency (i.e., the number of papers containing the keyword). This keyword appears in 1943 documents. The distribution of co-occurrence frequency and year of model is shown in Figure 2. Figure 2 shows that few researchers used the modeling method to study road risk prevention and control before 2010. After 2010, modeling research was favored by researchers. The number of papers on modeling research entered a rapid growth period after 2017.

Among all the papers related to “model”, the article named “Influence of Connected and Autonomous Vehicles on Traffic Flow Stability and Throughput” by Talebpour et al. has been widely concerned and recognized, with 422 citations (as of December 2021). A microsimulation model was set up in their work to study the stability of hybrid traffic flow composed of conventional vehicles, autonomous vehicles, and interconnected vehicles. Next, the influence of vehicle interconnection and automated driving technologies on the highway driving environment was revealed [42]. The study of Jia et al. also attracted the attention of researchers in the field. The authors defined the complex fleet system under the joint influence of the on-board random mobile network and the internal coordination mechanism of the fleet as a vehicular cyber-physical system (VCPS). They also thought that the queue-based vehicle dynamic control system was the close coupling between the dynamic characteristics of vehicles and the connection behavior of the vehicle network, which has a great impact on the formation and clustering of vehicles in VCPS [43]. Guo et al. studied the running behavior of bicycles at pedestrian crossings under red lights at signalized intersections and ordinary road sections. The results showed that the factors affecting the running behavior of bicycles under red lights mainly include gender, age, bicycle type, road width, separation width, signal type, green light rate, number of bicycles and vehicles, and average speed [44]. Rios-Torres et al. studied the coordination characteristics of networked and autonomous vehicles at intersections and the merging characteristics on the freeway ramps. They summarized the existing centralized and decentralized safety control methods in the industry to solve the traffic safety problems of intersections and ramps on the freeway [45]. In addition, seven highly cited papers are related to “model” [46,47,48,49,50,51,52]. 

The research topics of these papers were mainly about road traffic behavior prediction, traffic accident detection, vehicle control system, driving assistance system, and automated driving technology. Under the background of vehicle networking and automation technology, the interaction between driver and vehicle system is regarded as a key factor affecting driving safety, which belongs to active risk prevention and control research.

Since the 1960s, the concepts of active and passive safety have been used in the road transportation field. The development of vehicle engineering, electronic system, restraint system, airbag, and other devices installed in vehicles has greatly improved the safety performance of vehicles and ensured passive safety during driving. Therefore, active safety has become the key issue affecting driving safety and the focus of researchers. The research achievements in the field of vehicle active safety control system mainly include antilock braking system, electronic stability system, dynamic stability control system, driving assistance system, and automated driving technology. The driving assistance system mainly includes the assistance system of lane-keeping, automatic parking, and braking. The automatic driving system, which integrates risk prevention and control technologies, is a complex system engineering. Given that machine driving replaces human driving, automated driving technology considerably changes the existing road traffic operation state from the vehicle operating state, traffic flow characteristics, and other aspects. These problems should be the focus of future research.

Furthermore, the research on vehicle risk prevention and control technology needs interaction from drivers, traffic flow, and other driving environment conditions. The mathematical modeling can refine the perceptual and intuitive risk factors in the engineering practice, realize the quantitative study on the road engineering risk factors through the mathematical and physical models, and make the actual risk factors evolve within the preset range under the limited resource conditions. Hence, researchers can grasp the characteristics and effects of relevant risks in advance, and the solutions for the prevention and control of actual road traffic risks are provided. Therefore, the number of papers on model research has rapidly increased in the past decade.

#### 3.1.2. Analysis of “Risk”

**Figure 3:**
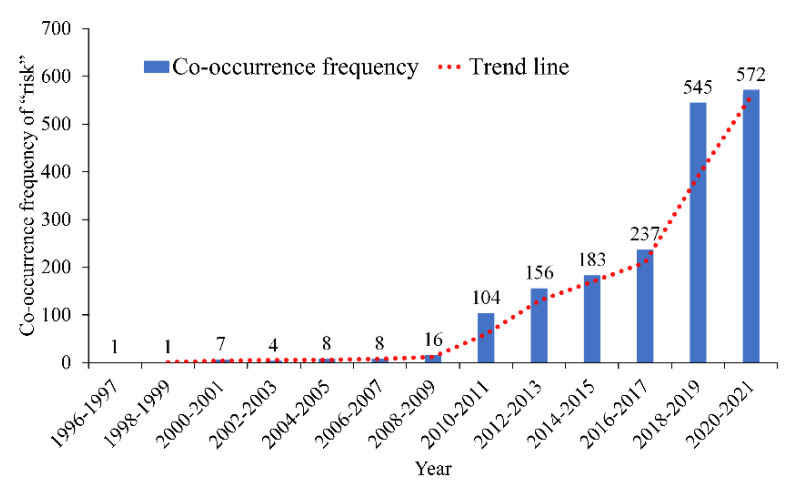
Co-occurrence frequency and time distribution of “risk”.

The co-occurrence frequency of the keyword “risk” is second only to “model”. The keyword “risk” appears in 1842 papers in the field of road traffic operation risk prevention and control. The distribution of co-occurrence frequency and year is shown in Figure 3. Figure 3 indicates that the number of research papers related to “risk” in the industry was negligible before 2010. After 2010, researchers began to pay considerable attention to the study of ”risk”. After 2017, the number of relevant papers entered a rapid growth period.

Among the 1842 papers related to “risk”, a study by Hulse et al. has been widely concerned and recognized. The authors investigated the subjects’ attitudes to road traffic risks under different traffic conditions and perspectives through an online questionnaire survey. They quantitatively studied the safety of self-driving vehicles from the perspective of gender, age, road users, and driving risks; their study made up for the lack of research perspective on automated driving technology in the industry [53]. Pregnolato et al. studied the impact of floods caused by extreme weather on the road traffic system. They established the relationship function between hydrostatic depth and driving speed; the maximum hydrostatic depth was proposed to ensure safe driving, parking, and steering through observation and measurement, thereby providing a reference for road flood risk analysis and transportation safety assessment under heavy rainfall conditions [54]. Foundas et al. analyzed the conventional road traffic accident data, weather data, and road surface information data by using the random parameter ordered probit model framework. They discussed the time-variant and time-invariant factors that affect the severity of single-vehicle accident injury. The results showed that the injury severity of single-vehicle accidents was affected by a series of time-variant (such as ice thickness, water depth, and surface temperature) and time-invariant (such as road geometry, vehicles, drivers, and collision characteristics) factors [55].

In addition, nine highly cited papers are related to “risk” [56,57,58,59,60,61,62,63,64]. The relative research topics are mainly about the drivers, driving behavior characteristics, traffic safety under adverse weather conditions, and influencing factors of traffic accidents. Among these topics, the research related to drivers and driving behavior characteristics occupied a large proportion. It belongs to the research area of human factor risks, including the following: traffic safety under adverse driving conditions, such as tunnel sections, long downhill sections, sharp bends, and other high-risk sections; driving safety under bad weather, such as rain, snow, and fog; driving hazards caused by the driver’s judgment, decision-making, and operation errors. All the aforementioned topics belong to human factor risk research. In traffic engineering, human factor risks are closely related to the physiological and psychological state of drivers, which are directly affected by the physical state and driving environment. Therefore, most of the existing studies in the industry usually take the physiological indicators of drivers when driving as the research point of penetration.

#### 3.1.3. Analysis of “Safety”

**Figure 4:**
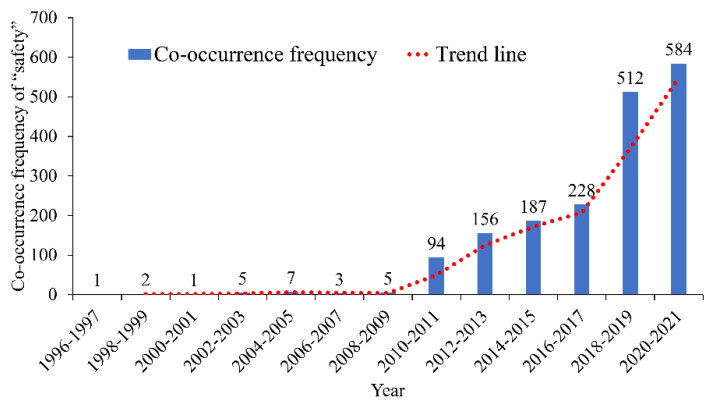
Co-occurrence frequency and time distribution of “safety”.

The co-occurrence frequency of the keyword “safety” is 1785 in the road traffic operation risk prevention and control field. The distribution of co-occurrence frequency and year is shown in Figure 4. Figure 4 indicates that the number of research papers related to “safety” in the industry was small before 2010. After 2010, the number of related papers increased appreciably and entered a rapid growth period.

Among the 1785 papers related to the keyword “safety”, the study by Li et al. has attracted wide attention, with 203 citations (as of December 2021). Their study used meta-analysis to evaluate the relationship between marijuana use and motor vehicle accident risk. The results showed that the use of marijuana by drivers is considerably related to the increase in motor vehicle collision risk; hence, their study provided a reference for the relevant research on the drug driving behavior of drivers [65]. Zhang et al. studied the problem of the integrated control system of automobile chassis; they proposed a flexible hierarchical structure scheme to realize self-tuning tire friction control and coordinated active steering and active wheel torque control that could adapt to road conditions [66]. Zheng et al. developed a distributed model predictive control algorithm for heterogeneous vehicle platoons with unidirectional topology and unknown expected set point [67]. Chen et al. established a short-term vehicle collision prediction model including weather information, road humidity, traffic speed, and traffic flow [68]. Moreover, four important studies are related to the keyword “safety” [69,70,71,72]. These studies mainly focused on high-risk driving behaviors, vehicle control systems, and vehicle collision predictions.

#### 3.1.4. Keyword Feature Analysis

The representative keywords of each period were extracted as follows by taking 5 years as the time interval:

From 1996 to 2000: Young driver, alcohol drinking, control motivations, physiological sensation, risk perception, cognitive concepts, automobile accident, safety, driving behavior, motor vehicle crash, speed, braking, elderly driver, drinking behavior, and risk assessment.

From 2001 to 2005: Driving risk, driver, tracking, road safety, random parameters, crash types, urban intersections, urban segments, system, identification, neural network, regression analysis, road vehicles, intelligent transportation systems, road traffic control, and driver information systems.

From 2006 to 2010: Mathematical model, stability analysis, human-driven vehicles, mixed traffic scenario, predictive control, nonlinear model, intelligent vehicle, sliding mode, braking system, prediction, behavior, machine learning, decision tree regression, statewide traffic analysis zone, ensemble technique, and brake reaction time.

From 2011 to 2015: Car-following model, road safety, weather, prediction model, risk-taking, broadcast, driving simulator, dynamical model, mental workload, vehicle collision, working memory, crash involvement, risk analysis, and geometric design.

From 2016 to 2021: Autonomous vehicle, electric vehicle, model predictive control, bicycle, security, driver injury severity, automated vehicle, UAV (unmanned autonomous vehicles), mobile phone use, crash frequency, ADAS (advanced driving assistance system), IoT (Internet of things), driver distraction, anger, V2X (vehicle-to-everything), support vector machine, V2V (vehicle-to-vehicle), single vehicle, older adult, traffic flow model, bridge, and gap acceptance.

On the basis of the keyword features in each period, the literature review, and our knowledge in the field, the international research progress can be summarized and analyzed as follows:

1. Drivers are the main risk source of road traffic accidents. Relevant research has been carried out in various periods.

The human factor risk is the focus of the research on the prevention and control of road traffic operation risk. In 1979, Treat et al. found that more than 90% of road traffic accidents are related to driver factors, and drivers and their driving behavior characteristics are the most important factors affecting road traffic safety [73]. The driver’s driving behaviors are closely related to their physiological and psychological states. The factors affecting driving behaviors are complex. These factors include weather characteristics, road alignment characteristics, traffic flow conditions, traffic ancillary facilities and their utility characteristics, driver’s understanding and compliance with traffic laws, and driver’s physical and mental health. Zhang et al. reviewed the international research on driving behavior and driving risks. The results showed that the research on driving risks mainly focuses on alcohol driving, drug driving, distracted driving, fatigue driving, and the driving behavior characteristics of young, old, and novice drivers [74]. 

Based on the above literature analysis, the present study believes that the existing research has ignored the impact of driving environment factors on the physiological and psychological characteristics of drivers and the related adverse driving behaviors to a certain extent. The impact of weather factors, traffic ancillary facilities, road alignment, and other factors on driving behaviors was also disregarded. These problems need further attention in future research.

The adverse weather environment has various effects on the physiological and psychological conditions of drivers. For example, the visual distance of drivers would decrease in rainy and snowy weather, and the driver’s psychological pressure would increase when judging driving conditions, resulting in an increase in driving load. Road driving conditions would deteriorate in rainy and snowy weather. In this case, road performance parameters, such as road surface friction coefficient reduction caused by water film or snow, would change. In this context, the driver’s driving decisions, such as speed selection and braking distance judgment, may not adapt to the driving environment and ensure safety. At present, researchers in the field have not paid enough attention to such safety issues.

The basic theories of road alignment design include vehicle dynamics and human factor engineering. In the road alignment design process, the human factors considered mainly include the driver’s reaction time, visual characteristics, and comfort level. In general, the safe operation of vehicles is ensured by the suitable combination of straight line and curve line indicators and the appropriate setting of road traffic auxiliary facilities. However, the rapid development of road engineering gradually exposes some traffic safety problems. For example, the researchers for the studies on the driving risk of long downhill road sections conducted various studies on the climbing performance of vehicles, such as the temperature change characteristics of brake drums. However, the driving behaviors of drivers on long downhill sections are ignored to a certain extent. The driver’s attention mechanism, judgment characteristics, and other physical and mental characteristics are closely related to their driving behaviors. These behaviors inevitably change when drivers drive on long downhill road sections with frequent braking, further affecting traffic safety. Therefore, these problems must be studied further in future research.

2. Vehicle collision, one of the road traffic risks, has received much attention from researchers because of its highest proportion of road traffic accidents.

The research on collision risk in the industry has never stopped. Previous research emphasized “take precautions” and mainly focused on analyzing vehicle collision risk factors, collision risk prediction, and collision avoidance technology. However, the research on traffic emergency response after collision accidents is insufficient and needs further improvement.

Effective emergency response measures can minimize the loss of life and property of drivers and passengers when a traffic accident occurs. The time for the injured to acquire timely rescue after a collision is critical. For a serious accident, a few minutes of delay would determine people’s lives and deaths. Therefore, reasonably planning the traffic accident rescue service stations must be reasonably planned, and timely rescue for the injured drivers and passengers when an accident occurs must be provided. Road emergency response technology is related to the humanistic care concept of road design and the layout of road traffic facilities and medical aid stations. These topics should be given considerable attention in future research to improve traffic safety.

3. The research on ITS has been gradually increasing in the 21st century.

The research on ITS began in the 1960s. As a professional term in the field of transportation, ITS was not officially recognized until the mid-1990s. Given the rapid development of Internet technology and data communication technology, the related research and application of ITS have gradually drawn considerable attention in the road traffic safety field since the beginning of the 21st century. Relevant research achievements in ITS mainly include vehicle control and management system, traffic monitoring and operation management system, public security traffic integrated command platform, traffic signal integrated control technology, and automated driving technology. 

The command platform integrated with public security traffic can use video monitoring and patrol inspection technology to monitor traffic events and judge traffic conditions, violations, and accidents. Moreover, this platform can track key vehicles, such as hazardous material transportation vehicles. The management and control technology integrated with traffic signals can be used in controlling road traffic restrictions and releasing traffic guidance information to provide necessary information for the safe operation of vehicles. Automated driving technology can avoid the human factor risk of vehicle operation to the maximum extent, and the safe and efficient operation of the road network can be realized using vehicle interconnection and vehicle-road cooperation technologies. ITS can only be realized on the basis of advanced communication, navigation, environmental perception, and identification technologies. 

In the ITS research, the connection between relevant technologies and the core issues of road traffic safety must be given attention to find the right place for the implantation of new technologies. Take the current development of the intelligent expressway as an example. One of the important objectives of developing intelligent expressways is to realize all-weather traffic for vehicles. The basic task of these intelligent expressways is to determine the characteristics of expressway traffic flow under different weather conditions. For example, the passenger and freight separation control technology in intelligent expressways plays an important role in ensuring the safety and rapid passage of vehicles. Differences exist in the dynamic performance, speed, steering, maneuverability, and driving behavior of passenger cars and trucks when passenger and freight transportation separation control measures in expressways are implemented. This scenario leads to variations in road traffic flow characteristics. In addition, compared with those in the hybrid traffic flow, the speed expectations of passenger car and truck drivers in expressways considerably change. In this case, the issues that need further evaluation and research include whether the conventional route that the technical indicators determined with speed as the core is compatible with the road safety facilities and whether the determination of the values of main technical indicators related to the construction scale and safety of the intelligent expressway is reasonable. The proper solutions to these problems are important foundations for the comprehensive construction of ITS.

4. The research related to topics such as autonomous vehicles, intelligent vehicles, V2V technology, V2X technology, and advanced driving assistance system in the field has increased appreciably since 2010.

The relevant keywords accounted for a large proportion of the research in the road traffic operation risk prevention and control field, whereas automated driving technology was the most concerning issue for researchers. Related research results were mainly distributed in the intelligent safety control of motor vehicles, vehicle adaptive cruise control, trajectory planning, driving assistance system, intelligent vehicle steering control, and other aspects [75,76,77,78]. Automated driving technology covers environmental cognition, decision-making planning, and vehicle control. Given that automated driving technology can replace human drivers, the road traffic operation risks caused by driver factors in different driving environments can be eliminated. 

Many aspects, such as road design and vehicle operation, would face new problems because of the upgrade and change of service objects. These problems include how the characteristics of road traffic flow would change in the context of vehicle networking and vehicle road cooperation technologies, how the road traffic flow would relate to road traffic safety, and how to improve the relevant standards of road alignment design under the traffic environment, where the proportion of human factor risk is reduced. These problems are closely related to road traffic safety and need further research. 

The current traffic development situation indicates that the application of high-level automated driving technology still has some time. In the future, the road traffic flow will inevitably experience a long-term “hybrid traffic flow” state, that is, the hybrid traffic state of different levels of automatic and conventional motor vehicles. In this case, considering the safety of hybrid traffic is also a problem that needs further research.

5. In the context of the noticeable increase in traffic congestion, low-carbon travel and green transportation have become the mainstream development direction. Moreover, the frequency of residents using nonmotor vehicles, particularly electric bicycles and shared bicycles, has increased appreciably. On the one hand, this change alleviates the problem of motor vehicle congestion. On the other hand, it exacerbates traffic safety problems, such as “pedestrian-vehicle conflict” and “vehicle-bicycle conflict.” These traffic conflicts are more serious at urban road intersections than they are on other types of roads. This concern should be given considerable attention in future research.

As shown in Table 1, the future research directions are summarized from the above analysis.

### 3.2. Literature Review of CSCD

**Figure 5:**
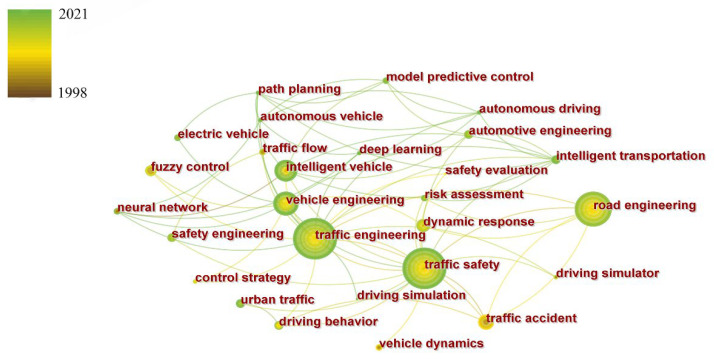
Co-occurrence network of partial high-frequency keywords in CSCD.

From 7876 relevant papers in CSCD, 2255 keywords meeting the threshold conditions were extracted by CiteSpace. Figure 5 shows the co-occurrence network of partial high-frequency keywords in CSCD.

According to the co-occurrence frequency, the top 30 keywords in 23 years from 1998 to 2021 are as follows:

Traffic engineering, traffic safety, road engineering, expressway, vehicle engineering, simulation, driving behavior, intelligent transportation, traffic accidents, urban traffic, automatic driving, fuzzy control, numerical simulation, vehicles, bridge engineering, safety engineering, highway tunnel, model predictive control, intelligent vehicle, electric vehicle, traffic flow, genetic algorithm, cellular automata, vehicle networking, tunnel engineering, control strategy, driver, risk assessment, intersection, and traffic conflict.

#### 3.2.1. Analysis of “Traffic Engineering”

**Figure 6:**
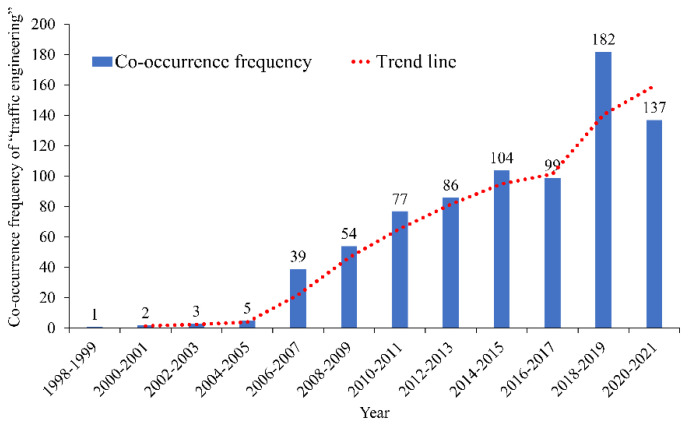
Co-occurrence frequency and time distribution of “traffic engineering”.

Among the keywords extracted by CiteSpace, “traffic engineering” has the highest co-occurrence frequency. It appeared in 789 papers in the road traffic operation risk prevention and control field. The distribution of co-occurrence frequency and year is shown in Figure 6. 

Figure 6 indicates that the studies related to “traffic engineering” in the industry before 2006 are few. The number of relevant research gradually increased after 2006 and entered a rapid growth period after 2016. Related research involved many topics among which the drivers and their driving behavior characteristics have received extensive attention from researchers in the industry. 

For example, Zheng et al. studied the driver’s visual characteristics under free traffic flow conditions; they established the mathematical model of the driver’s preview time in straight and curved sections [79]. Feng et al. established a driver’s “perception decision control behavior” model based on the hidden Markov model. Among all kinds of driving behaviors, vehicle-lane-changing behavior, including its risk, is one of the most concerned directions of researchers in the industry [80]. Wang et al. established three kinds of lane-changing safety identification models to explore the safe vehicle distance under different lane-changing situations [81]. Moreover, researchers gave considerable attention to the driving risks at high-risk road sections, such as tunnels, curved slopes, bridges [82,83], and intersections [84].

#### 3.2.2. Analysis of “Traffic Safety”

**Figure 7:**
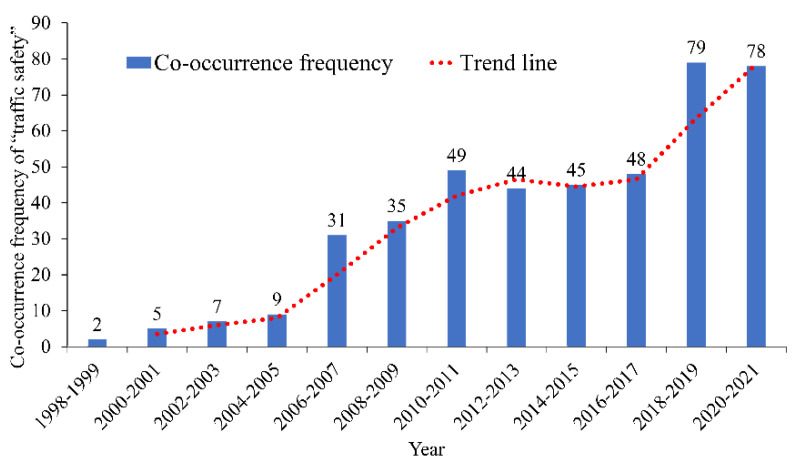
Co-occurrence frequency and time distribution of “traffic safety”.

The co-occurrence frequency of the keyword “traffic safety” is 432, only second to “traffic engineering”. The distribution of co-occurrence frequency and year is shown in Figure 7. 

Figure 7 shows that the studies related to “traffic safety” in the industry before 2006 are few. The number of relevant papers entered a rapid growth period after 2016. Driver characteristics and driving behavior risks are the most important research directions related to “traffic safety”. For example, Shan et al. studied the influence of roadside billboards on drivers’ attention; they identified that the layout density of roadside billboards is the main factor affecting the attention of drivers [85]. Shi et al. studied the steering operation and lane departure characteristics of a driver under the visual distraction condition; the results showed that the driver’s operation on the steering wheel gradually decreases with the increase in visual distraction time, whereas the degree of lane departure gradually increases [86]. For various adverse weather conditions, the researchers paid more attention to road traffic safety on foggy days than that on rainy and snowy days [87,88].

#### 3.2.3. Analysis of “Road Engineering”, “Expressway”, and “Vehicle Engineering”

**Figure 8:**
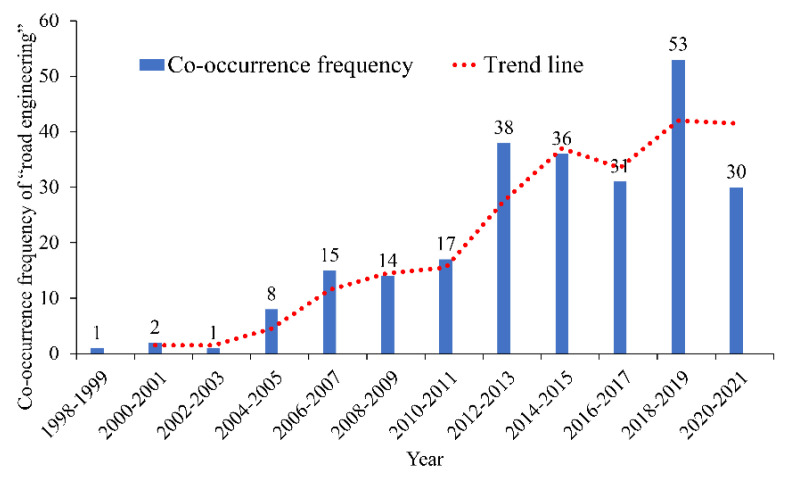
Co-occurrence frequency and time distribution of “road engineering”.

**Figure 9:**
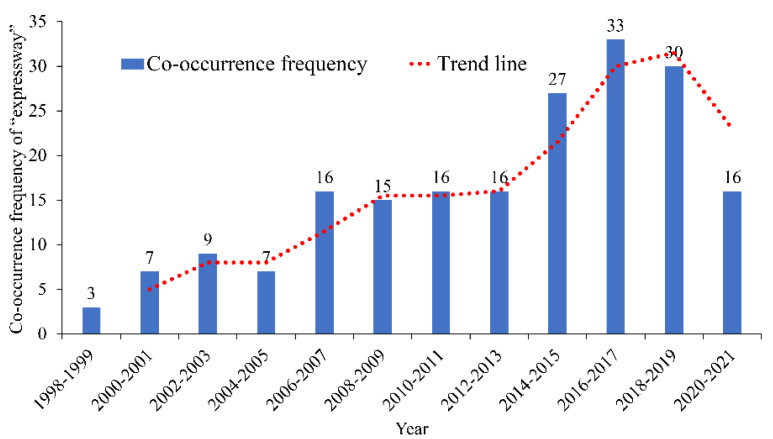
Co-occurrence frequency and time distribution of “expressway”.

**Figure 10:**
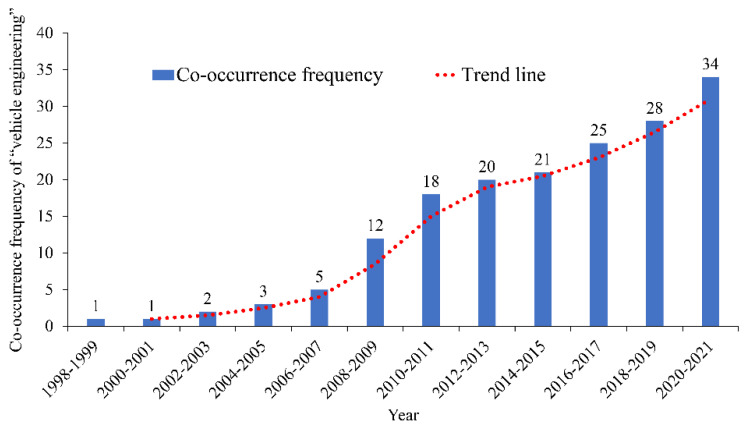
Co-occurrence frequency and time distribution of “vehicle engineering”.

The co-occurrence frequencies of the keywords “road engineering”, “expressway”, and “vehicle engineering” are 246, 195, and 170, respectively. These keywords rank third to fifth. The relationships between the co-occurrence frequencies and the corresponding years are shown in Figure 8, Figure 9 and Figure 10. The analysis results show that before 2006, the number of research papers related to “road engineering” in the industry was small; after 2006, it increased considerably, showing an increasing trend. The number of research papers related to “expressway” reached the peak between 2018 and 2019, whereas that of research papers related to “vehicle engineering” increased annually.

The safety of road alignment design [89,90,91] has been widely concerned by researchers in the research related to “road engineering” and “expressway”. The research results involved many aspects, such as the design of roundabouts, longitudinal slopes, and hedging lanes. For example, Meng et al. studied the characteristics and prediction problems concerning the driving speed on the combined sections of the horizontal curve and longitudinal slope of the expressway; they also established a running speed prediction model [89]. Xu et al. studied the influence of clothoid on the driving speed on the curve; the results showed that the setting of the clothoid increases the track radius and driving speed of the vehicles on the curve [90]. 

In addition, the safe road driving speed is another critical topic. Feng et al. studied the influence of the coupling of environmental illumination and speed change on the driver’s visual function through real vehicle tests; they determined the road speed limit under dynamic low illumination [92]. Wang et al. set up a variable speed limit control model of expressway based on delay and accident loss; they also proposed the calculation method of comprehensive loss of road section [93]. Furthermore, some researchers analyzed the vehicle rollover risk and rear-end collision risk [94,95].

The research results of the keyword “vehicle engineering” were mainly related to the vehicle control system and algorithm. For example, Niu et al. built a whisker algorithm for intelligent vehicle route cruising and autonomous obstacle avoidance to realize autonomous driving and reasonable obstacle avoidance [96]. Moreover, the research on the rollover risk of vehicles and hazardous material transportation vehicles [97,98] and the studies on the prevention and control of vehicle collision and lane-changing risk [99,100] attracted the attention of researchers to a certain extent. For example, Li et al. studied the lateral stability of the closed-loop system of semitrailer trains. They established a driver model with time delay. They found that reducing the driver’s reaction delay time and keeping proper driving load and running speed obviously affect the improvement of the lateral stability of semitrailer trains [97]. Gao et al. studied the driver’s visual behavior under different vehicle speeds through real vehicle-lane-changing experiments [99]. In addition, a small number of studies include the automatic driving field [101].

Based on the comprehensive analysis of the abovementioned high-frequency keywords and the research results in the related papers, the research topics of road traffic operation risk prevention and control in China in the past 20 years can be summarized as follows:

1. Human factor risks: Drivers and their driving behavior characteristics, including driver’s attention mechanism, visual characteristics, driving style, and operating behavior.
2. Vehicle factor risks: Vehicle speed control and safety, including safe vehicle operating speed, speed limit under various driving conditions, safety and stability of vehicle control system, control algorithm of the vehicle under various working conditions, prevention and control of vehicle rollover, collision, and lane-changing risks, and the safety of automated driving technology.
3. Road factor risks: Driving risks of high-risk road sections, including tunnels, curved slope sections, bridges, and intersections; the safe design of road alignment, such as the index design of longitudinal slope sections.
4. Environmental factor risks: Driving risks under adverse weather conditions, of which the driving risk on foggy days is the most concerned, followed by the driving risks on rainy and snowy days.
5. Others: Characteristics and influencing factors of road traffic accidents.

#### 3.2.4. Keyword Feature Analysis

The representative keywords of each period were extracted as follows by taking 5 years as a time interval:

From 1999 to 2003: Traffic engineering, traffic safety, urban roads, entrance and exit traffic, interchanges and elevated roads, highways, simulation, road engineering, vehicle engineering, driving behavior, road tunnel, path planning, vehicle dynamics, driver model, BP neural network, lane-keeping, driver, intelligent traffic system, vehicle safety, and conflict probability.

From 2004 to 2008: Bridge engineering, tunnel engineering, traffic conflict, car-following model, signal intersection, safety engineering, risk assessment, analytic hierarchy process, risk analysis, stability control, hazardous material transportation, driver behavior, unmanned driving, regression analysis, electric-assisted steering, intelligent traffic system, tunnel entrance and exit, intelligent transportation, model test, and lane-changing.

From 2009 to 2013: Transportation system engineering, tunnel lighting, driving safety, expressway tunnel, emergency evacuation, joint simulation, rollover, multiobjective optimization, response time, long downhill, hazardous material transportation, model predictive control, Bayesian network, ride comfort, differential braking, interchange, real vehicle test, vehicle road coordination, dynamic planning, urban expressway, and nonmotor vehicles.

From 2014 to 2018: Internet of vehicles, double-layer planning, wheel motor, driving assistance system, hierarchical control, stability analysis, cumulative prospect theory, horizontal curve section, natural driving, trajectory planning, visual characteristics, machine learning, lane-changing behavior, anti-rollover control, driverless vehicle, cooperative adaptive cruise control, control science and technology, automatic driving vehicle, collision time, automatic emergency braking, artificial intelligence, V2V communication, and risk identification.

From 2019 to 2021: Intelligent networked vehicles, semantic segmentation, driving safety, artificial bee colony algorithm, collaborative optimization, attention mechanism, traffic information, advanced driving assistance system, intelligent networked vehicles, longitudinal control, intelligent networked vehicles, information physical system, driving simulation test, dangerous driving behavior, grassland roads, lane line identification, extra-long tunnels, collision warning, driving conditions, simulation platform, and unconventional intersection.

The prevention and control of road traffic operation risk require all-round and multilevel means rather than single or partial technical measures. From the perspective of drivers, vehicles, roads, and the environment, the research on road traffic system safety in China is generally comprehensive. It involves most risk factors of drivers, vehicles, roads, and the environment by considering risk prevention and control both at the macro and micro scales. However, the existing research results and the actual situation of road traffic safety in China show problems that need further solutions. Based on the keyword features in each period, the literature review, and our knowledge in the field, the research progress in China can be summarized and analyzed as follows:

1. All the high-frequency keywords set for each period involve the research topic of driver characteristics and driving behaviors. This finding indicates that driver factors are usually the leading causes of accidents. Accident statistics also highlight the importance of human factors to road system design.

The current research analyzed various physiological and psychological characteristics and dangerous driving behaviors from the perspective of drivers and driving behaviors. However, effective means for solving problems in the industry are currently unavailable. These problems include taking appropriate measures and technologies in specific road traffic to monitor accurately and warn the high-risk driving status of drivers while driving, capturing the dangerous driving behaviors, and correcting risky behaviors. In addition, the current driver training system in China mainly emphasizes the evaluation of the participants’ physiological conditions, such as screening individuals who are color blind and testing eyesight and hearing conditions. However, this evaluation does not investigate the psychological conditions of driving test personnel, and the follow-up driving test training plans are not formulated accordingly.

For example, different driving test training strategies should be developed for drivers with medical personality attributes of type A, B, or C. After the issuance of the driving license, the management and assessment systems for drivers do not consider their risky operations and dangerous driving behaviors. Although the dangerous driving behaviors of drivers do not cause traffic accidents in a short time, these behaviors are potential risks to road traffic. Once an accident occurs, a considerable loss of life and property may occur. Therefore, relevant risks must be prevented and controlled from the source. Moreover, the process of taking a driving test to obtain a license must be recorded. Further research on the above issues should be considered in the future.

2. In the past decade (from 2010 to 2021), the research on vehicle engineering has increased appreciably. It has become the research focus of road traffic operation risk prevention and control in China. The rapid development of science and technology has led to the transformation of vehicle engineering. New vehicle traffic technologies, mainly represented by vehicle-to-infrastructure cooperation systems, vehicle networking, automatic driving, and V2V, belong to the research hotspots in the road traffic safety field.
3. From the perspective of vehicle operation monitoring and management, the problems needing further research in the future are as follows: screening and monitoring potential accident vehicles and dangerous operating vehicles from the huge and dense real-time vehicle data of road traffic flow; issuing an early warning and taking timely control measures against the target vehicles; adequate supervision of the whole process of transportation vehicles for hazardous materials and special materials.
4. In road design work in China, the tolerant design concept is underappreciated to some extent.

For drivers, the increase in driving mileage may lead to an increase in the probability of errors in judgment and operation when driving. The tolerant design concept refers to the design decisions of “avoiding traffic accidents” and “reducing the severity of accidents.” Given the mistakes that drivers may make, designers should take these decisions in the road design stage to reserve safety space for the faulty operation of drivers.

At present, the safety evaluation of the design indicators in road design in China is still based on meeting the relevant standards and specifications. The formulation of these standards and specifications is based on a driver’s reasonable judgment and driving operation. Moreover, the possible driving errors are not fully considered. Therefore, the specific implementation of the tolerant design concept, such as road alignment guidance design, subgrade slope control design, design of roadside buffer and energy dissipation facilities, and traffic guidance sign design, must be given considerable attention during road design and construction. This approach, combined with road traffic practice experience, can achieve remarkable technological innovation and breakthroughs.

5. The research on traffic safety in tunnel sections is a critical issue throughout the history of road traffic operation risk prevention and control research.

Tunnels have unique structures and limited internal space. They are also characterized by closed spaces, high noise, sudden changes in lighting at the entrance and exit, complex traffic organization conditions, poor air circulation, and other adverse traffic factors. Thus, the environment inside a tunnel seriously affects drivers’ driving psychology and behaviors.

Currently, the research on tunnel traffic safety mainly focuses on driver characteristics and driving behavior, tunnel lighting technology, and traffic signs. When an accident occurs in a tunnel, the impact area is wide, and the duration is long. Thus, conducting traffic diversion and rescue is challenging. In future research, establishing a full chain response mechanism of an accurate early warning system, efficient rescue in an accident, and rapid recovery after the accident must be considered. Furthermore, the problem of drivers’ cognition of tunnel safety facilities must be solved, and the key technology for the effective setting of tunnel traffic safety facilities must be realized.

6. The risk prevention and control of hazardous material transportation vehicles is an important task for traffic management departments and transportation enterprises. However, the relevant studies on this topic are few.

Hazardous materials mainly include explosives, compressed gas, and liquefied gas. These materials are generally flammable, explosive, toxic, and corrosive; thus, they are critical hidden dangers for road traffic safety. Once an accident occurs to such vehicles, the secondary damage caused by the leakage, combustion, and explosion of the hazardous materials consequentially impairs road network safety and the ecological environment. The severity of this kind of traffic accident, particularly in special sections such as tunnels, bridges, and mountain roads, is far greater than that of traffic accidents caused by ordinary vehicles.

At present, the safety supervision of hazardous material transportation vehicles in China mainly includes supervising the public security traffic management departments and the self-management of transportation enterprises. Relevant departments ensure the safety of hazardous materials transportation vehicles mainly from the aspects of special vehicle restrictions, cargo loading, storage requirements, onboard safety facilities assembly, driver qualification requirements, special vehicle custody, transportation route planning, vehicle positioning tracking, and driver status monitoring. Some regions have taken measures to restrict the traffic of hazardous material transportation vehicles. In practice, these measures for ensuring traffic safety are difficult to implement fully and effectively because of the lack of responsibility awareness of transportation enterprises, the limitation of vehicle monitoring technology, the limited resources of traffic management departments, and the inadequate network supervision and management system. 

For example, as dynamic risk sources, hazardous material transportation vehicles must be tracked in real time, and the safety status of vehicles must be evaluated timely. However, real-time tracking of these vehicles on the road network in remote areas and the prompt monitoring and warning of drivers are difficult because of the inadequacy of road informatization, network monitoring, and management in China.

Moreover, specific road environment poses a great threat to the safety of hazardous material transportation vehicles. For example, during night transportation, hazardous material transportation vehicles are prohibited from taking the expressway, and most cities prohibit them from entering urban areas. However, effective safety management for the national and provincial roads and other sections in suburban areas is lacking, thereby posing a threat to road safety. In some areas, drivers of hazardous material transportation vehicles often exhibit speeding, fatigue driving, and distracted driving behaviors because of the lack of surveillance and speed measurement systems and the light traffic flow on the road at night. These behaviors are potential traffic risks and should be specially studied. In addition, hazardous material transportation in long downhill road sections, bridges, and tunnels and transportation under adverse weather conditions are high-risk transportation types that need further research.

7. Similar to hazardous material transportation vehicles, overlimit transportation vehicles and their safety management have not gained enough attention.

Overlimit transportation refers to trucks that exceed the specified length, width, and height of road construction gauges or trucks with a total load exceeding the load limit standard of roads and their structures. Overlimit transportation vehicles must submit an application to the traffic management departments before transportation. They can only go on the road after approval. The safety management problem of such vehicles has not been properly solved in China.

In recent years, many traffic accidents caused by overloaded transportation vehicles that lead to the collapse of overpasses have occurred from time to time, resulting in irreparable loss of life and property of drivers and passengers. The safety management and risk prevention and control of overlimit transportation vehicles should be carried out from the aspects of risk assessment, transport mileage limitation, transportation route planning, transportation process monitoring, and traffic organization and guidance. However, the monitoring and management of overlimit transportation vehicles in China exhibit many loopholes and deficiencies, such as the monitoring and control of these vehicles when they pass through low-grade roads, overpasses, and tunnels. In summary, further research should be conducted on the risk control and safety guarantee issues of overlimit transportation vehicles.

8. Research on emergency rescue for road traffic emergencies needs to be further improved.

Active and effective emergency rescue response is vital to reducing the loss of life and property of drivers and passengers in traffic accidents. The Ministry of Transport of China has successively issued Emergency Plan for Highway Transportation Accidental Event [102], Emergency Plan for Road Transportation Accidental Event [103], Emergency Plan for Urban Bus and Trolley Bus Accidental Event [104], and other documents for road traffic emergency rescue. The emergency rescue response involves a series of technical problems, such as the road network control plan in an emergency, the rapid planning plan for rescue vehicle routes, the traffic guidance plan for avoiding secondary accidents, and the medical rescue service plan for injured people. Thus, the related departments must decide quickly according to the collected information within a limited time. The layout and resource allocation of emergency rescue stations, road information management, and rescue of special sections (tunnels, bridges, and separated subgrade sections) must be considered in the road design and construction stages. These issues must be further studied to lay a theoretical foundation for rapid and effective emergency rescue.

As shown in Table 2, the future research directions are summarized from the above analysis.

## 4. Discussion

Based on bibliometrics, this study analyzed the literature on road traffic operation risk prevention and control from WoSCC and CSCD in the past 26 years by using CiteSpace software. Based on the systematic research of a large number of papers in the field, this study summarized the research context, hotspots, and noteworthy achievements in the road traffic operation risk prevention and control field in the past 26 years from the perspective of keyword co-occurrence analysis. Given the analysis results and the current situation of road traffic safety, the problems that have not been given enough attention and need to be further solved were put forward.

The contents of the entire paper indicate that our review research has at least three shortcomings:

1. The databases selected in the study are relatively limit. Only WoSCC and CSCD were selected as the literature retrieval databases. The literature that is not written in English and Chinese was excluded.
2. Only the first few keywords with high co-occurrence frequency were analyzed.
3. The studies on the road traffic operation risk prevention and control field from different countries were not analyzed comparatively in this study.

In future research, the aforementioned shortcomings of this work will be considered.

## 5. Conclusions

The worldwide research progress of road traffic operation risk prevention and control was comprehensively analyzed, with the literature from WoSCC and CSCD as research materials. Moreover, the research results were summarized, and the problems to be further solved were put forward.

1. In terms of research methods, studying the prevention and control of road traffic operational risk by establishing models is the mainstream research mode. “Model”, “risk”, and “safety” are the three most occurred keywords from the English literature; “traffic engineering”, “traffic safety”, “road engineering”, “expressway”, and “vehicle engineering” are the five most occurred keywords from the Chinese literature. Relevant research accounts for a large proportion of the literature on road traffic operation risk prevention and control.
2. Research on road traffic operation risk prevention and control was mainly conducted from the perspective of drivers, vehicles, roads, and the environment.

At the level of human risk prevention and control, related research mainly focused on drivers and their driving behavior characteristics, such as the characteristics of alcohol driving, drug driving, fatigue driving, distracted driving, elderly drivers, and novice drivers. 

At the level of vehicle risk prevention and control, the research results were mainly distributed in the vehicle control system, driving assistance system, hazardous material transportation vehicles, automated driving technology, safe driving speed, and vehicle collision prediction. 

At the level of road risk prevention and control, relevant research mainly focused on the traffic safety guarantee of high-risk road sections, such as bridges, tunnels, and intersections, and the safety of road alignment design. 

At the level of environmental risk prevention and control, road traffic safety under adverse weather was the research focus.

3. Researchers have not paid enough attention to the emergency response to road traffic emergencies. The issues that need further research include the following: the road network traffic control scheme under emergencies; the planning and lay-out of road emergency rescue facilities; the traffic diversion and rescue during emergencies in tunnels; the full chain response mechanism of an accurate early warning system, efficient rescue in events, and rapid recovery after the event of tunnel traffic; the effective setting technology of tunnel traffic safety facilities.
4. The detection and early warning of driver’s high-risk driving status, the improvement of driver training mechanism and management system, the monitoring of vehicle operation, the safety management of hazardous material transportation, and the safety control of overlimit transportation vehicles must be further studied. Targeted technical measures must be proposed to ensure the safety of road traffic operations.
5. Research on the impact of driving environment factors on the physiological and psychological characteristics of drivers and the related risky driving behaviors, such as the relationship between weather factors, traffic ancillary facilities, road alignment, and driving behaviors, must be further promoted.
6. The research results of automated driving technology mainly focused on environmental perception and identification, vehicle positioning and path planning, lane selection, and speed control. The integration of automated driving technology with conventional road design theory and driving dynamics theory and the safety of “hybrid traffic” formed by automatic driving vehicles and conventional motor vehicles must be researched further.
7. Researchers in China have ignored the “tolerant design concept” in road traffic planning and design to a certain extent. Thus, the corresponding research must be strengthened in the future.

## Figures and Tables

**Table 1:** Future research directions based on WoSCC.

	Future Research Directions	Specific Examples
1	The impact of driving environment factors on the physiological and psychological characteristics of drivers and adverse driving behaviors	The influence of adverse weather environment on physiological and psychological conditions of drivers; driving risks on long downhill sections
2	Traffic emergency response after accidents	Planning of traffic accident rescue service station; humanistic care concept in road design; optimization of layout of road traffic facilities
3	The connection between ITS technologies and the core issues of road traffic safety	Expressway traffic flow characteristics under different weather conditions; variation of expressway traffic flow characteristics under the condition of passenger and freight transportation separation
4	Changes in road traffic flow characteristics due to service object upgrading	Changes in road traffic flow characteristics in the context of vehicle networking and vehicle-road cooperation technologies
5	Various traffic conflicts on urban roads	Traffic conflicts between non-motor vehicles and motor vehicles; traffic conflicts between pedestrians and motor vehicles; traffic conflicts between pedestrians and non-motor vehicles

**Table 2:** Future research directions based on CSCD.

	Future Research Directions	Specific Examples
1	Effective technologies for driving risk monitoring	Technologies for monitoring and warning drivers of high-risk driving conditions, capturing dangerous driving behaviors, and correcting dangerous behaviors
2	Optimization of driver training system	Investigating the psychological status of driving test personnel and developing the corresponding training strategies
3	Screening and monitoring of potential accident vehicles and dangerous operating vehicles	Screening and monitoring potential accident vehicles and dangerous operating vehicles from the huge and dense real-time vehicle data of road traffic flow
4	Tolerant design concept in road design work	Road alignment guidance design; subgrade slope control design; roadside buffer and energy dissipation facilities design; traffic guidance sign design
5	Risk prevention and control of tunnel sections	Response mechanism of an accurate early warning system, efficient rescue in the accident, and rapid recovery after the accident
6	Risk prevention and control of hazardous material transportation vehicles	Supervising the whole process of hazardous material transportation vehicles; risk prevention and control technology of hazardous material transportation vehicles operating on roads at night; safety guarantee technology for hazardous material transportation vehicles in long downhill sections, bridges, tunnels, and other sections; monitoring and management technology of hazardous material transportation vehicles under bad weather conditions
7	Safety management of overlimit transportation vehicles	Risk assessment, transport mileage limitation, transportation route planning, transportation process monitoring, and traffic organization and guidance of overlimit transportation vehicles
8	Emergency rescue for road traffic emergencies	Road network control plan in an emergency; rapid planning plan for rescue vehicle routes; traffic guidance plan for secondary accident prevention; medical rescue service plan for injured people; layout and resource allocation of emergency rescue stations, road information management, and rescue of special sections (tunnels, bridges, and separated subgrade sections) in the road design and construction stages

## Data Availability

Not applicable.
